# Comment on von Känel et al. Early Trauma-Focused Counseling for the Prevention of Acute Coronary Syndrome-Induced Posttraumatic Stress: Social and Health Care Resources Matter. *J. Clin. Med.* 2022, *11*, 1993

**DOI:** 10.3390/jcm11206036

**Published:** 2022-10-13

**Authors:** Ivana Sopek Merkaš, Nenad Lakušić

**Affiliations:** 1 Department of Cardiology, Special Hospital for Medical Rehabilitation Krapinske Toplice, 49217 Krapinske Toplice, Croatia; 2 Department of Clinical Medicine, Faculty of Dental Medicine and Health Osijek, 31000 Osijek, Croatia; 3 Department of Internal Medicine, Family Medicine and History of Medicine, Faculty of Medicine Osijek, 31000 Osijek, Croatia

**Keywords:** acute coronary syndrome, posttraumatic stress disorder, cardiovascular disease

## Abstract

Posttraumatic stress disorder (PTSD) is a debilitating disorder, and it is known that it can be triggered by acute coronary syndrome (ACS). Patients with ACS-induced PTSD have an increased risk of recurrent adverse cardiovascular events and mortality. This is still an insufficiently recognized subgroup of patients among clinicians that could benefit from specific therapeutic and rehabilitation approaches.

We have read with great interest the article by von Känel et al. [1]. Posttraumatic stress disorder (PTSD) following an acute coronary syndrome (ACS) is an increasingly recognized and described entity, but the incorporation of its diagnosis and treatment into daily clinical practice is still deficient and not standardized. These patients have an increased risk of recurrent adverse cardiovascular events and mortality. This secondary analysis [1] of the data collected as a part of the MI-SPRINT randomized controlled trial [2] concluded that an early psychological intervention after ACS with a trauma-focused approach for patients who perceive high social support or participate in cardiac rehabilitation (CR) for several weeks may be beneficial in the prevention of PTSD symptoms’ development, which may be clinically relevant. In the primary trial [2], the patients were randomized to one single session of early trauma-focused counseling or stress-focused counseling, thus establishing an active comparator design. The results of this article [1], although interesting and promising, requires replication given the exploratory nature of the analyses.

The prevention of PTSD symptoms and early intervention may also be useful in the context in which the physical setting where the treatment is initiated (i.e., the emergency department) can be perceived as particularly stressful and likely to contribute to the development of PTSD symptoms [3]. There is currently insufficient evidence from randomized controlled trials that early interventions to prevent the development of PTSD after ACS are effective [4], and this secondary analysis [1] asserts social and health care resources of traumatized patients as moderators of treatment outcome. In daily clinical practice there are increasingly more patients with PTSD symptoms seen in CR and it is important to recognize such patients to provide adequate treatment. They are less adherent to medications [5] and if experiencing cardiac symptoms (chest tightness/pain, heartbeat symptoms) often avoid physical activity [6], both of which are possible mechanisms in negative cardiovascular outcomes of these patients.

CR is an important aspect of care for patients after ACS. According to our experience in a rehabilitation center, patients with PTSD symptoms after ACS are still an insufficiently recognized subgroup of patients [7]. Gaining support and knowledge, learning how to cope with the symptoms, psychological support, and optimal drug therapy are indispensable in the process of improving the outcome and quality of life of patients with ACS-induced PTSD. Support from psychologists/psychiatrists and cardiologists during CR is very important and may have an impact on patients who have experienced ACS, and these effects need further investigation [1]. A team of cardiologists and psychologists/psychiatrists and their intervention can be crucial in preventing the development of PTSD. Since this special subgroup of patients should be recognized first, we believe that a standardized screening for PTSD symptoms at the beginning of rehabilitation should be a part of the treatment process in order to provide a specific therapeutic rehabilitation approach that should result in the improvement of the quality of life and reduction in the overall mortality of such a vulnerable group of patients.
